# Chronic Psychosocial Stress in Mice Is Associated With Increased Acid Sphingomyelinase Activity in Liver and Serum and With Hepatic C16:0-Ceramide Accumulation

**DOI:** 10.3389/fpsyt.2018.00496

**Published:** 2018-10-16

**Authors:** Martin Reichel, Cosima Rhein, Lena M. Hofmann, Juliana Monti, Lukasz Japtok, Dominik Langgartner, Andrea M. Füchsl, Burkhard Kleuser, Erich Gulbins, Claus Hellerbrand, Stefan O. Reber, Johannes Kornhuber

**Affiliations:** ^1^Department of Psychiatry and Psychotherapy, Friedrich-Alexander-Universität Erlangen-Nürnberg, Erlangen, Germany; ^2^Department of Nephrology and Medical Intensive Care, Charité – Universitätsmedizin Berlin, Berlin, Germany; ^3^Institute of Nutritional Sciences, University of Potsdam, Nuthetal, Germany; ^4^Laboratory for Molecular Psychosomatics, Clinic for Psychosomatic Medicine and Psychotherapy, University of Ulm, Ulm, Germany; ^5^Department of Internal Medicine I, University Hospital Regensburg, Regensburg, Germany; ^6^Department of Molecular Biology, University of Duisburg-Essen, Essen, Germany; ^7^Institute of Biochemistry, Emil-Fischer-Zentrum, Friedrich-Alexander-Universität Erlangen-Nürnberg, Erlangen, Germany

**Keywords:** chronic psychosocial stress, acid sphingomyelinase, ceramide, sphingolipid metabolism, chronic subordinate colony housing (CSC), liver metabolism

## Abstract

Chronic psychosocial stress adversely affects human morbidity and is a risk factor for inflammatory disorders, liver diseases, obesity, metabolic syndrome, and major depressive disorder (MDD). In recent studies, we found an association of MDD with an increase of acid sphingomyelinase (ASM) activity. Thus, we asked whether chronic psychosocial stress as a detrimental factor contributing to the emergence of MDD would also affect ASM activity and sphingolipid (SL) metabolism. To induce chronic psychosocial stress in male mice we employed the chronic subordinate colony housing (CSC) paradigm and compared them to non-stressed single housed control (SHC) mice. We determined Asm activity in liver and serum, hepatic SL concentrations as well as hepatic mRNA expression of genes involved in SL metabolism. We found that hepatic Asm activity was increased by 28% (*P* = 0.006) and secretory Asm activity by 47% (*P* = 0.002) in stressed mice. C16:0-Cer was increased by 40% (*P* = 0.008). Gene expression analysis further revealed an increased expression of tumor necrosis factor (TNF)-α (*P* = 0.009) and of several genes involved in SL metabolism (*Cers5, P* = 0.028; *Cers6, P* = 0.045; *Gba, P* = 0.049; *Gba2, P* = 0.030; *Ormdl2, P* = 0.034; *Smpdl3B*; *P* = 0.013). Our data thus provides first evidence that chronic psychosocial stress, at least in mice, induces alterations in SL metabolism, which in turn might be involved in mediating the adverse health effects of chronic psychosocial stress and peripheral changes occurring in mood disorders.

## Introduction

Stress, defined as the physiological response of the body to any demand (1), serves the principal goal to mobilize energy for appropriate, fight or flight' response. The primary response includes the activation of the sympathetic nervous system and the hypothalamus-pituitary-adrenal axis, resulting in the secretion of catecholamines and glucocorticoids from the adrenal gland. Albeit this adaptation promotes survival of physical threats to homeostasis, chronic psychosocial threats are well-known to adversely affect human health (2–4).

Chronic psychosocial stress, particularly in conjunction with viral hepatitis, cirrhosis, and hepatocellular carcinoma is thought to contribute to the development and progression of liver disease (5). For instance, a comparison of the mortality in the general population between periods of economic crisis and periods prior or after the crisis revealed an increased all-cause mortality, due among others to an increased incidence of chronic liver disease (3). Moreover, chronic stress is a well-known risk factor for the development of obesity and metabolic syndrome (6, 7). Animal studies further support the hypothesis that chronic stress induces (8) and aggravates (9, 10) liver injury, causes hepatic oxidative stress (11, 12) and insulin resistance (13), alters hepatic metabolism and gene transcription (14), and disrupts the regulation of lipid synthesis (15).

Sphingolipids (SL) comprise a class of lipids with important structural functions and relevance in cell signaling (16). Bioactive SL play a role in the regulation of cell growth, death, senescence, adhesion, migration, inflammation, angiogenesis, and intracellular trafficking (17). Ceramides (Cer) constitute a family of lipid species (18) that are central to SL metabolism as they serve as precursors for the biosynthesis of plasma membrane SL such as glycosphingolipids (GSL) or sphingomyelins (SM), and, alternatively, can be metabolized into other bioactive SL such as ceramide-1-phoshate, sphingosine, and sphingosine-1-phosphate. Additionally, Cer modulate a number of biochemical and cellular processes induced by stressor exposure, including apoptosis, cell-cycle arrest and cell senescence. Moreover, several extracellular challenges, such as tumor necrosis factor (TNF)-α, chemotherapeutic agents and heat, cause Cer accumulation (19). Increased Cer levels, in turn, are supposed to contribute to the development of several human diseases, including liver diseases (20). Cer can be synthesized *de novo* from serine and palmitoyl-CoA or by re-acylation of sphingosine in a salvage pathway. Cer can be also generated by the breakdown of SL from biological membranes. Acid sphingomyelinase (here referred to as ASM for human protein and Asm for mouse protein) is one of several mammalian sphingomyelinases that catalyzes the breakdown of SM to Cer and phosphorylcholine (21). Activity of ASM is sensitive to cellular stress and is activated, among others, by TNF-α (22), oxidative stress (23), and ionizing radiation (24). In line with these findings, recent studies provided evidence that both stress-associated disorders, as for instance major depression (25), chronic heart failure (26), acute and chronic alcohol consumption (27, 28), and chronic hepatitis C infection (29), as well as various chronic unpredictable stressors (25, 30), have been associated with increased ASM activity and Cer levels, respectively.

However, it is unknown to date whether this holds true for chronic stressors which are psychosocial in nature. We therefore analyzed mice subjected to the chronic subordinate colony housing (CSC) paradigm, a pre-clinically validated mouse model for chronic psychosocial stress (31). Importantly, besides typical stress symptoms such as adrenal hypertrophy, thymus atrophy, and increased plasma nor-epinephrine levels, CSC exposure reliably causes anxiety and spontaneous colitis, increases the risk for colon cancer (32), and induces hepatic inflammation and oxidative stress (11). Our analysis of serum samples and liver specimen revealed that chronic psychosocial stress is associated with increased Asm activity, increased levels of C16:0-Cer, a decline in C24:0-Cer, and increased expression levels of *Cers5, Cers6, Gba, Gba2, Ormdl2*, and *Smpdl3b* mRNA. Thus, a shift in the SL composition toward an accumulation of C16:0-Cer might be the origin of the adverse health effects associated with chronic psychosocial stress.

## Methods

### Animal specimens

Liver and serum specimens of male C57BL/6 mice (Charles River, Sulzfeld, Germany) that were either exposed to CSC (*n* = 8) for 19 days or kept as SHC (*n* = 8) were analyzed. Animals and procedures are described in detail in Czech et al. (11). Briefly, four CSC mice were housed together with a larger dominant male in a polycarbonate observation cage (38 × 22 × 35 cm) for 19 d consecutively. Prior to CSC exposure, all potential male dominant mice were tested for their aggressive behavior. Males that started to injure their opponents by harmful bites were not used for the CSC procedure. To avoid habituation, each dominant male was replaced by a novel dominant male on days 8 and 15. Serum of independent CSC and SHC groups (each *n* = 8) was used to confirm results. All experimental protocols were approved by the Committee on Animal Health and Care of the Government of Oberpfalz (Permit Number: 54-2531.2-16/08) and conform to international guidelines on the ethical use of animals.

### Preparation of tissue lysates and determination of Asm activity

Asm activity was determined from liver homogenates and from serum. Tissue, homogenates and serum were stored at −80°C prior to the analysis. For the preparation of liver homogenates, pieces of 10–20 mg tissue were homogenized in 0.5 ml sucrose lysis buffer (250 mM sucrose, 1 mM EDTA, 0.2% Triton X-100) using a TissueLyser LT bead mill (Qiagen). Raw lysates were centrifugated with ≥10,000 g at 4 °C for 10 min, and supernatants were transferred to new tubes. The protein concentrations were determined using bicinchoninic acid kit (Sigma). For the determination of Asm activity from liver homogenates, 1 μg of protein were incubated with 0.58 μM *N*-(4,4-difluoro-5,7-dimethyl-4-bora-3a,4a-diaza-s-indacene-3-dodecanoyl)-sphingosylphosphocholine (BODIPY® FL C_12_-sphingomyelin; D-7711; Life Technologies, Darmstadt, Germany) in 50 μl reaction buffer (50 mM sodium acetate pH 5.0, 0.3 M NaCl, 0.2% NP-40) for 2 h at 37°C; after incubation, 3 μl of the reaction volume was spotted on a silica gel 60 plate (Macherey-Nagel; Düren, Germany), and Cer and SM were separated by thin layer chromatography using 99% ethyl acetate/1% acetic acid (v/v) as a solvent (33). Intensity of BODIPY-conjugated Cer and SM fractions were determined using a Typhoon Trio scanner (GE Healthcare, München, Germany) and quantified with QuantityOne software (Biorad, München, Germany). For each liver specimen, Asm activity was determined twice from two independent pieces of tissue with similar results. Activity of secretory (S-) Asm was measured with the same protocol using 2 μl of serum and with additional 500 μM ZnCl_2_ in the reaction buffer.

### Quantification of ceramide and sphingomyelin species by mass spectrometry

Cer and SM were extracted and quantified as described previously (25). Briefly, lipid extraction was performed from 100 μl liver homogenates containing 50 μg total protein using C17:0-Cer and deuterated C16-d31 SM (N-palmitoyl-d31-D-*erythro*-sphingomyelin; Avanti Polar Lipids) as internal standards. Sample analysis was carried out by rapid-resolution liquid chromatography-MS/MS using a Q-TOF 6,530 mass spectrometer (Agilent Technologies, Waldbronn, Germany) operating in the positive ESI mode. The precursor ions of Cer species [C16:0-Cer (m/z 520.508), C17:0-Cer (m/z 534.524), C18:0-Cer (m/z 548.540), C20:0-Cer (m/z 576.571), C22:0-Cer (m/z 604.602), C24:0-Cer (m/z 632.634), C24:1-Cer (m/z 630.618)] were cleaved into the fragment ion m/z 264.270. The precursor ions of SM species [C16:0-SM (m/z 703.575), C16-d31 SM (m/z 734.762), C18:0-SM (m/z 731.606), C20:0-SM (m/z 759.638), C22:0-SM (m/z 787.669), C24:0-SM (m/z 815.700), C24:1-SM (m/z 813.684)] were cleaved into the fragment ion m/z 184.074. Quantification was performed with Mass Hunter Software (Agilent Technologies).

### Extraction of RNA and synthesis of cDNA

Total RNA was isolated from pieces of liver tissues (<30 mg) using a TissueLyser LT bead mill (Qiagen) and peqGOLD Trifast reagent (Peqlab, Erlangen, Germany) according to manufacturers' instructions. RNA qualities and concentrations were assessed using a Nanodrop ND-1000 UV-Vis spectrophotometer. SuperScript VILO cDNA synthesis kit (Invitrogen) was used to reverse transcribe 1 μg RNA into cDNA using 2 μl 5x VILO reaction mix and 1 μl 10x SuperScript enzyme mix in a final volume of 10 μl. After completion and termination of the RT reaction, cDNA was diluted with 190 μl LowTE and stored at −20°C.

### Quantitative PCR analysis

Quantitative real-time PCR was performed using a LightCycler 480 real-time PCR system (Roche, Germany) and SYBR-green chemistry. In detail, qPCR reactions contained 5 μl FastStart Essential DNA Green Master, 1 μM of each primer and 2.5 μl diluted cDNA (corresponding to 12.5 ng RNA) in a total volume of 10 μl. Temperature profile used was: 95°C for 5 min followed by 45 cycles of amplification (95°C for 10 s, 60°C for 20 s, 72°C for 30 s) and by melting curve analysis. After run, PCR product specificity was assessed by the inspection of single peak melting curves, and threshold cycles (Ct) were determined with the second derivative maximum method using the LightCycler 480 software (release 1.5.0). Gene-specific primers were either selected from the literature or from PrimerBank (https://pga.mgh.harvard.edu/primerbank/) or designed via the Universal Probe Library Assay Design Center (http://qpcr.probefinder.com/organism.jsp). Reference genes were tested and selected according to their gene expression stability (34), and a normalization factor was calculated based on the geometric mean of the reference genes *Rpl32, Rpl38, Hprt*, and *Gusb* (34) using the SLqPCR package in R version 3.2.2. Relative mRNA expression levels were calculated in Microsoft Excel using the 2^−ΔΔCt^ method (35). The sequence of primers is given in Table 1.

**Table 1:** Sequence of primers used in this study.

**No**.	**MGI Symbol**	**Gene name**	**RefSeq**	**Fwd primer**	**Rev primer**
1	Asah1	N-acylsphingosine amidohydrolase 1	NM_019734	5′-TGAAGATGGTGGATCAAAAGC-3′	5′-ACATCTGCAATTCCCCTCA-3′
2	Asah2	N-acylsphingosine amidohydrolase 2	NM_018830	5′-TTCTCACCCTCTTGTTTGTTACC-3′	5′-AGGGAAGTTTGGAGTCTGTGT-3′
3	Cerk	Ceramide kinase	NM_145475	5′-TCCGTGCTGTGGGTGAAAC-3′	5′-CGCAGTCGTCTTTTTCCTCAA-3′
4	Cers1	Ceramide synthase 1	NM_138647	5′-CCACCACACACATCTTTCGG-3′	5′-GGAGCAGGTAAGCGCAGTAG-3′
5	Cers2	Ceramide synthase 2	NM_029789	5′-AGAGTGGGCTCTCTGGACG-3′	5′-CCAGGGTTTATCCACAGTGAC-3′
6	Cers3	ceramide synthase 3	NM_001164201	5′-CCTGGCTGCTATTAGTCTGATG-3′	5′-CTGCTTCCATCCAGCATAGG-3′
7	Cers4	Ceramide synthase 4	NM_026058	5′-CTGTGGTACTGTTGTTGCATGAC-3′	5′-GCGCGTGTAGAAGAAGACTAAG-3′
8	Cers5	Ceramide synthase 5	NM_028015	5′-GACTGCTTCCAAAGCCTTGAG-3′	5′-GCAGTTGGCACCATTGCTAG-3′
9	Cers6	Ceramide synthase 6	NM_172856	5′-GGAGCTGTCATTTTATTGGTCTTT-3′	5′-GGAACATAATGCCGAAGTCC-3′
10	Galc	Galactosylceramidase	NM_008079	5′-CGCCTACGTGCTAGACGAC-3′	5′-ACGATAGGGCTCTGGGTAATTT-3′
11	Gba	Glucosidase, beta, acid	NM_008094	5′-TTTGGTAAAGCACCTCGGTATG-3′	5′-GCATGTCGATGAAAGGGGTCT-3′
12	Gba2	Glucosidase beta 2	NM_172692	5′-TTTGGTAAAGCACCTCGGTATG-3′	5′-GCATGTCGATGAAAGGGGTCT-3′
13	Ormdl1	ORM1-like 1	NM_145517	5′-ACAGTGAGGTAAACCCCAATACT-3′	5′-GCAAAAACACATACATCCCCAGA-3′
14	Ormdl2	ORM1-like 2	NM_024180	5′-CCTGGAGACCACAGGTGTAAG-3′	5′-AGCCCTGATTGAGCTTGTTC-3′
15	Ormdl3	ORM1-like 3	NM_025661	5′-ACCCTCACCAACCTTATCCA-3′	5′-GTCAGCAACCTTGCTTTGC-3′
16	Sgms1	Sphingomyelin synthase 1	NM_001168525	5′-GAGCTGTGACCTTTTGAGCA-3′	5′-TTATATCCAGTTGCCCCTGTG-3′
17	Sgms2	Sphingomyelin synthase 2	NM_028943	5′-TTACCTGTGCCCGGAATG-3′	5′-TTTGCCTGAGAGTCTCCATTG-3′
18	Sgpl1	Sphingosine phosphate lyase 1	NM_009163	5′-CTGAAGGACTTCGAGCCTTATTT-3′	5′-ACTCCACGCAATGAGCTGC-3′
19	Sgpp1	Sphingosine-1-phosphate phosphatase 1	NM_030750	5′-TACGGGCTGATTCTCATTCCC-3′	5′-GGTCCACCAATGGGTAGAAGA-3′
20	Sgpp2	Sphingosine-1-phosphate phosphotase 2	NM_001004173	5′-TCTACCATGGACCGGTATCAG-3′	5′-GAGACACACCAGCGTAGAGAAC-3′
21	Smpd1	Sphingomyelin phosphodiesterase 1, acid lysosomal	NM_011421.	5′-TGCTGAGAATCGAGGAGACA-3′	5′-GACCGGCCAGAGTGTTTTC-3′
22	Smpd3	Sphingomyelin phosphodiesterase 3	NM_021491	5′-TCTACCTCCTCGACCAGCAC-3′	5′-TGCTGCTCCAGTTTGTCATC-3′
23	Smpdl3a	Sphingomyelin phosphodiesterase, acid-like	NM_020561	5′-TCCTTTGCTGCCTACTGGTT-3′	5′-TCAGTCACGTGCCAAAACTG-3′
24	Smpdl3b	Sphingomyelin phosphodiesterase, acid-like 3B	NM_133888	5′-TTGTGGAACGCTTGACCAAC-3′	5′-GAACTGGTTCTTAGGGTGGAAG-3′
25	Sphk1	Sphingosine kinase 1	NM_011451	5′-GGTGAATGGGCTAATGGAACG-3′	5′-CTGCTCGTACCCAGCATAGTG-3′
26	Sphk2	Sphingosine kinase 2	NM_203280	5′-TCTGGAGACGGGCTGCTTTA-3′	5′-TCAAACCCGCCATGATGGTTC-3′
27	Sptlc1	Serine palmitoyltransferase, long chain base subunit 1	NM_009269	5′-ACGAGGCTCCAGCATACCAT-3′	5′-TCAGAACGCTCCTGCAACTTG-3′
28	Sptlc2	Serine palmitoyltransferase, long chain base subunit 2	NM_011479	5′-AACGGGGAAGTGAGGAACG-3′	5′-CAGCATGGGTGTTTCTTCAAAAG-3′
29	Sptlc3	Serine palmitoyltransferase, long chain base subunit 3	NM_175467	5′-TCTGAACGACAGTGCTGTTAC-3′	5′-ATGCCTTCCTATTTTGCTGGG-3′
30	Ugcg	UDP-glucose ceramide glucosyltransferase	NM_011673	5′-GGAATGGCCTTGTTCGGCT-3′	5′-CGGCTGTTTGTCTGTTGCC-3′
31	Ugt8a	UDP galactosyltransferase 8A	NM_011674	5′-TCAGAAGACATTGCCAACAAA-3′	5′-GGTTCTTTGGTTTGGTTCCAG-3′
33	Tnf	Tumor necrosis factor	NM_013693	5′-CTGTAGCCCACGTCGTAGC-3′	5′-TTGAGATCCATGCCGTTG-3′
34	Hmox1	Heme oxygenase 1	NM_010442	5′-AGGCTAAGACCGCCTTCCT-3′	5′-TGTGTTCCTCTGTCAGCATCA-3′
35	Bmp4	Bone morphogenetic protein 4	NM_007554	5′-GAGGAGTTTCCATCACGAAGA-3′	5′-GCTCTGCCGAGGAGATCA-3′
37	Gusb	Glucuronidase, beta	NM_010368	5′-GATGTGGTCTGTGGCCAAT-3′	5′-TGTGGGTGATCAGCGTCTT-3′
38	Gapdh	Glyceraldehyde-3-phosphate dehydrogenase	NM_008084	5′-AGGTCGGTGTGAACGGATTTG-3′	5′-TGTAGACCATGTAGTTGAGGTCA-3′
39	Hprt	Hypoxanthine guanine phosphoribosyl transferase	NM_013556.	5′-CCTCCTCAGACCGCTTTTT-3′	5′-AACCTGGTTCATCATCGCTAA-3′
40	Rpl32	Ribosomal protein L32	NM_172086	5′-TTAAGCGAAACTGGCGGAAAC-3′	5′-TTGTTGCTCCCATAACCGATG-3′
41	Pmm1	Phosphomannomutase 1	NM_013872	5′-GTCCTGGCGGGAATGACTTT-3′	5′-TGGGCTGTCTCTGGGAAGAA-3′
42	Rpl38	Ribosomal protein L38	NM_001048057	5′-AGGATGCCAAGTCTGTCAAGA-3′	5′-TCCTTGTCTGTGATAACCAGGG-3′

### Statistical analyses

Statistical analyses were performed using the IBM SPSS Statistics version 21. Continuous variables were tested for deviation from normal distributions using the Kolmogorov–Smirnov test. Statistical significance of measurements between SHC and CSC mice was determined using ANOVA. A two-sided *P* < 0.05 was considered to indicate statistical significance. All results are presented as mean value ± standard deviation (SD). Graphs were created with GraphPad Prism for Windows, Version 4.01 (Graphpad Software, LaJolla, CA) or with MS Excel 2010.

## Results

### Asm activities are elevated in stressed mice

**Figure 1:**
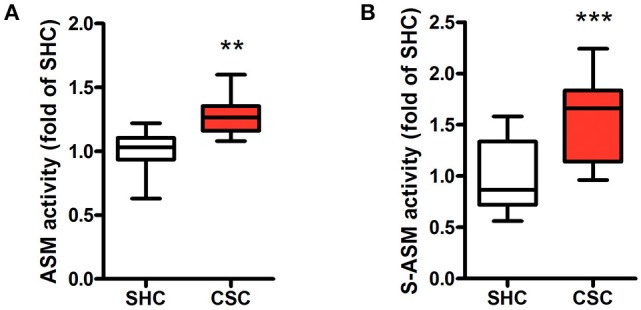
Chronic psychosocial stress is associated with increased Asm activity in liver and serum. Asm activity in liver **(A)** and serum **(B)** of SHC and CSC mice (each *n* = 8 for liver and *n* = 16 for serum). For ease of comparison, activities of SHC mice was set to 1. Data are presented as mean values ± SD. Asterisks indicate statistical significance CSC vs. SHC (**p < 0.01, ***p < 0.0001). CSC, chronic subordinate colony housing; SHC, single housed controls.

Hepatic Asm activity in CSC mice was increased by 28% in comparison to SHC mice (Figure 1A; relative Asm activity in SHC: 1.00 ± 0.18, *n* = 8; CSC: 1.28 ± 0.17, *n* = 8; *P* = 0.006). We also determined activity of S-Asm in serum. S-Asm activity of CSC mice was increased by 57% (Figure 1B; relative S-Asm activity in SHC: 1.00 ± 0.34, *n* = 16; CSC: 1.57 ± 0.37, *n* = 14; *P* = 0.0001).

### Stressed mice display higher percentage of C16:0-ceramide

**Figure 2:**
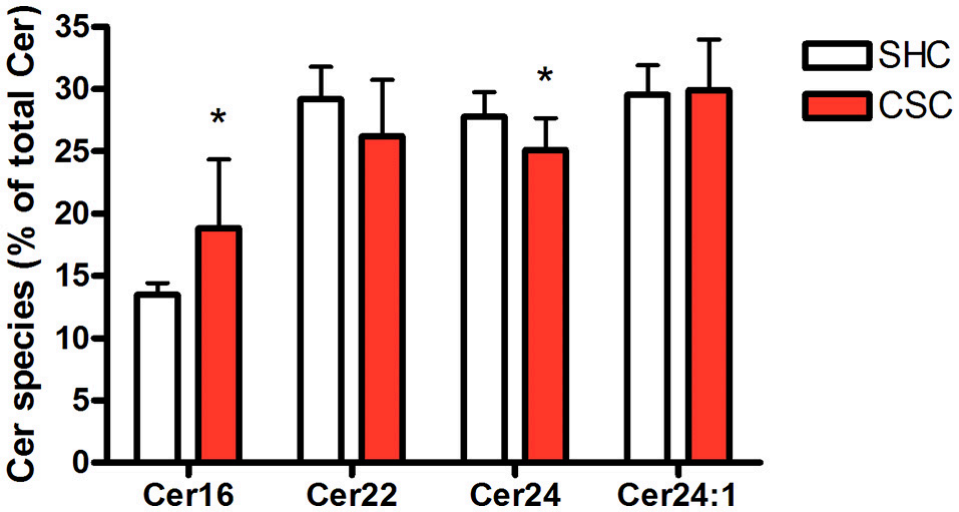
Chronic psychosocial stress is associated with increased C16:0-Cer and decreased C24:0-Cer content in the liver. Given is the percentage of each Cer species with respect to total Cer quantity. Data are presented as mean values ± SD (*n* = 8 per group). Asterisks indicate statistical significance CSC vs. SHC (*p < 0.05). Cer, ceramides; CSC, chronic subordinate colony housing; SM, sphingomyelines; SHC, single housed controls.

Hepatic tissue was subjected to lipidomic analyses to determine Cer and SM content. Total Cer (SHC: 643 ± 63.4 pmol/mg protein, *n* = 8; CSC: 640 ± 68.9 pmol/mg protein, *n* = 8; *P* = 0.927) and SM levels (SHC: 2,320 ± 239 pmol/mg protein, *n* = 8; CSC: 2.677 ± 440 pmol/mg protein, *n* = 8; *P* = 0.064) were not increased in CSC mice. To assess the relative composition of the hepatic Cer pool, we calculated the relative percentage for every single species. The composition of the Cer pool was changed significantly in response to stress toward a higher percentage of long chain C16:0-Cer (from 13.5 to 18.8%, *P* = 0.017) and a lower percentage of Cer species with very-long saturated acyl chains (C22:0-Cer: 29.2 to 26.2%, *P* = 0.127; C24:0-Cer: 27.8 to 25.1%, *P* = 0.033) (Figure 2).

### TNF-α mRNA expression is upregulated in CSC mice

We further analyzed TNF-α mRNA expression levels in liver tissue. In agreement with a previous report (11), TNF-α mRNA was significantly increased in CSC compared to controls (SHC: 1.00 ± 0.42, *n* = 8; CSC: 2.43 ± 1.15, *n* = 8; *P* = 0.009).

### RNA expression of SL metabolizing enzymes is upregulated in CSC mice

**Figure 3:**
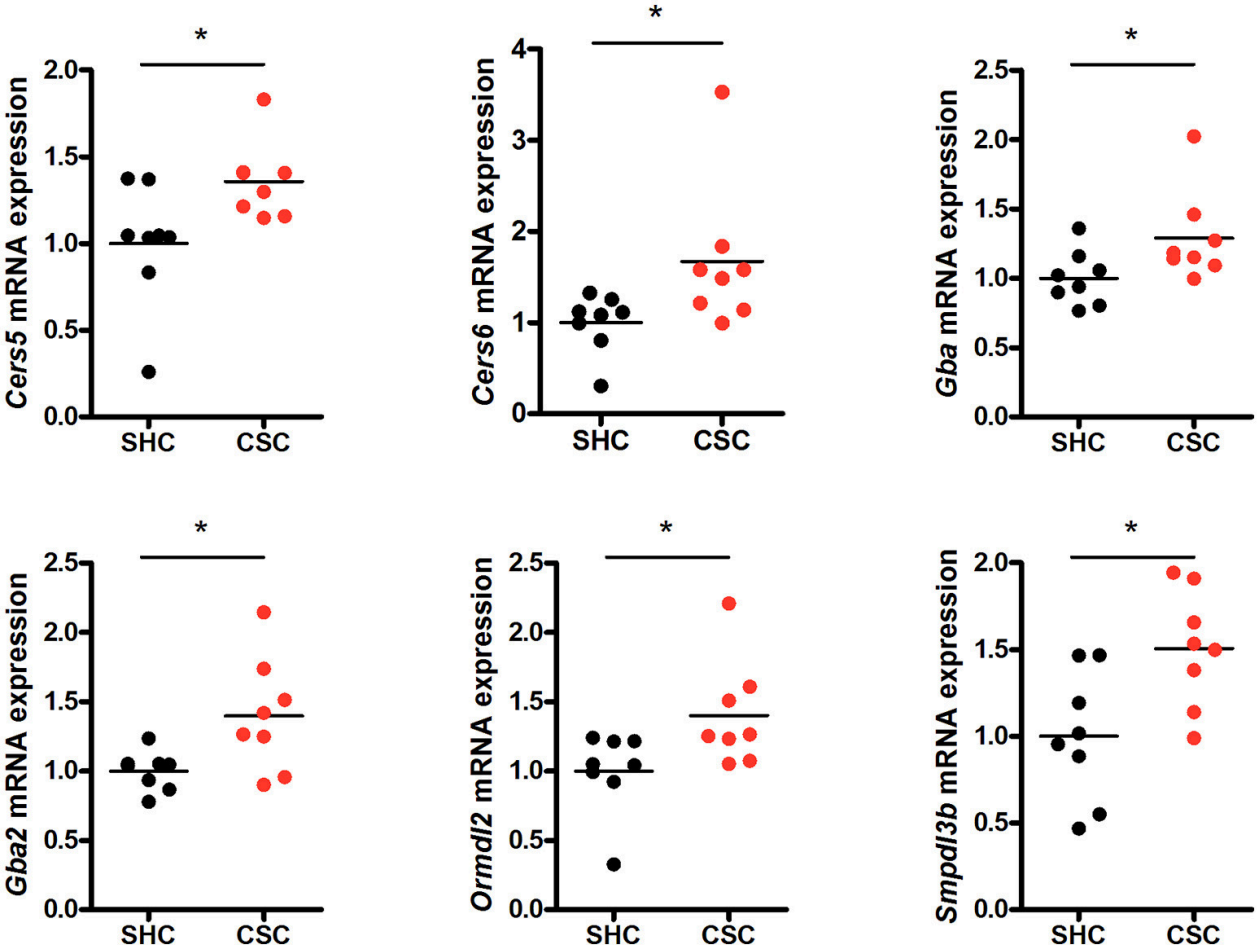
Chronic psychosocial stress is associated with altered gene expression in the liver of enzymes regulating ceramide production. Relative mRNA expression of genes involved in sphingolipid metabolism. Vertical scatter plot of genes differentially expressed in CSC vs. SHC (*t-*tests, *P* < 0.05). Selection of reference genes and calculation of the normalization factor was conducted according to Vandesompele et al. (34). For ease of comparison, transcript level in SHC mice was set to 1. Asterisks indicate statistical significance CSC vs. SHC (*p < 0.05). CSC, chronic subordinate colony housing; SHC, single housed controls.

To check if other pathways in addition to the SM hydrolyzing pathway via Asm were involved in the change of C16:0-Cer levels upon chronic psychosocial stress, we conducted a gene expression analysis of 31 genes involved in the metabolism of Cer in liver tissue (Table 1). Three genes, ceramide synthase 1 (*Cers1*), ceramide synthase 3 (*Cers3*), and serine palmitoyltransferase, long chain base subunit 3 (*Sptlc3*) were not expressed in hepatic tissue (*data not shown*). A comparison of the normalized mRNA expression levels of the other 28 genes revealed that the majority was higher expressed in CSC compared to SHC mice (Table 2). *Cers5, Cers6, Gba, Gba2, Ormdl2*, and *Smpdl3b* mRNA were significantly higher expressed in CSC mice compared to control mice (Figure 3).

**Table 2:** Gene expression analysis of genes involved in SL metabolism.

**MGI symbol**	**Change (%)**	***P*-value**
Asah1	3.88	0.719
Asah2	−11.2	0.423
Cerk	16.1	0.297
Cers2	−25.2	0.162
Cers4	−11.6	0.554
**Cers5**	**35.9**	**0.028**
**Cers6**	**66.9**	**0.045**
Galc	16.9	0.162
**Gba**	**28.9**	**0.049**
**Gba2**	**39.7**	**0.030**
Sgms1	20.7	0.198
Sgms2	−4.79	0.756
Sgpl1	−0.85	0.965
Sgpp1	−1.88	0.911
Sgpp2	11.7	0.656
Smpd1	32.8	0.075
Smpd3	90.5	0.130
Sphk1	24.5	0.385
Sphk2	−11.4	0.315
Sptlc1	24.0	0.086
Sptlc2	49.7	0.052
Ugcg	18.3	0.220
Ugt8a	−2.95	0.879
Ormdl1	0.95	0.954
**Ormdl2**	**39.9**	**0.034**
Ormdl3	−0.32	0.984
Smpdl3a	22.9	0.344
**Smpdl3b**	**50.7**	**0.013**

*Significance of differentially expressed genes under chronic psychosocial stress was estimated using t-test. Genes with P < 0.05 are given in bold*.

## Discussion

In this study, we showed for the first time that chronic psychosocial stress in mice leads to the activation of Asm and, consequently, increased Cer levels, both assessed in hepatic tissue. In detail, increased Asm activity in mice exposed to chronic psychosocial stress was associated with increased concentration of C16:0-Cer and a decline of C24:0-Cer. Increased C16:0-Cer concentrations and especially a shift from very-long-chain C24:0-Cer to long chain C16:0-Cer in the SL composition is frequently observed (36) and confers susceptibility to cellular apoptosis (37) and steatohepatitis and insulin resistance (38). Thus, activation of Asm seems likely to constitute an important link between chronic psychosocial stress and its adverse health effects in the liver, but also in other organs.

Our analysis further revealed that chronic psychosocial stress affects other key aspects of sphingolipid regulation besides activation of ASM. By means of gene expression analysis we found that several enzymes involved in SL metabolism—*Cers5* and *Cers6, Gba, Gba2, Ormdl2*, and *Smpdl3b—*were upregulated under conditions of chronic psychosocial stress. Ceramide synthase (CerS) 5 and 6 (MGI symbol *Cers5* and *Cers6*) belong to a group of enzymes that catalyze the formation of ceramides by *N*-acylation of sphingoid bases. Each CerS has a high specificity toward the acyl chain length and, thus, the active CerS isozymes determine the fatty acid composition of Cer and the derived SL (39). Of the six mammalian CerSs we found four—*CerS2, CerS4, CerS5*, and *CerS6—*to be expressed in hepatic mouse tissue, while we could not detect transcripts of *CerS1* or *CerS3*. These results are in line with a previous report (40). Increased *CerS5* or *CerS6* activity can mediate the accumulation of C16:0-Cer (41, 42). Of note, several reports indicate that *CerS6* activity is controlled via transcriptional regulation (42, 43). Thus, increased expression of *CerS5* and *CerS6* following chronic psychosocial stress might be responsible for the shift in the cellular SL composition from very-long-chain Cer to long-chain C16-Cer and, thus, contribute to the adverse health effect of stress. However, since CerS are also regulated by post-translational mechanisms (44, 45), enzymatic activities of *CerS5* and *CerS6* under conditions of chronic psychosocial stress should to be determined before claiming a role in stress-induced health issues.

We also found two glucosylceramidases, *Gba* and *Gba2*, to be transcriptionally upregulated under chronic psychosocial stress. This could also contribute to increased Cer levels, resulting from the hydrolysis of glucosylceramide. Chronic psychosocial stress also induced increased expression *Ormdl2. Ormdl2* belongs to the evolutionarily conserved family of ORM-like proteins, which are central regulators of SL metabolism (46). *Ormdl* proteins are negative regulators of *de novo* ceramide synthesis via inhibition of serine palmitoyltransferase (SPTLC), the first and rate-limiting enzyme in SL production. However, *Ormdl* proteins can also stimulate *de novo* synthesis of complex SL downstream of SPTLC in yeast (47) via a mechanism that potentially involves CerS activity (48). Sphingomyelinase-like phosphodiesterase 3b (MGI symbol: *Smpdl3b*) is a GPI-anchored SM phosphodiesterase with relevance for cellular lipid composition (49). Knockdown of *Smpdl3b* in RAW264.7 macrophages results in decreased Cer level, indicating that the protein acts as an enzyme and generates Cer.

In summary, our analysis provides first evidence that chronic psychosocial stress has an impact on the hepatic SL metabolism in mice. Whether this relates to the primary stress response, e.g., secretion of glucocorticoids and catecholamines,—not mutually exclusively—or to the strong connection of SL with inflammation and oxidative stress (50) needs to be determined in future studies. Similar changes of the SL metabolism might be involved in the development of stress-associated pathologies in humans, and key enzymes of SL metabolism such as ASM (51) and *CerS6* (52) might constitute therapeutic targets to prevent or treat such conditions.

## Author contributions

MR and SR conceived and designed the experiments. MR and CR wrote the manuscript. MR, LH, JM, LJ, BK, DL, and AF performed experiments. MR, CR, JK, and EG analyzed the data. CH contributed reagents, materials, and analysis tools. EG and JK provided funding. All authors reviewed the manuscript.

### Conflict of interest statement

The authors declare that the research was conducted in the absence of any commercial or financial relationships that could be construed as a potential conflict of interest. The handling Editor declared a shared affiliation, though no other collaboration, with one of the authors MR. The reviewer KB declared a shared affiliation, with no collaboration, with several of the authors DL, AF, and SR to the handling Editor.
